# Targeting the Vulnerabilities of Oncogene Activation

**DOI:** 10.3390/cancers15133359

**Published:** 2023-06-27

**Authors:** Christina Huang, Jack L. Arbiser

**Affiliations:** 1 Sidney Kimmel Medical College, Thomas Jefferson University, Philadelphia, PA 19107, USA; cxh503@students.jefferson.edu; 2 Metroderm/United Dermatology Partners, 875 Johnson Ferry Rd., Atlanta, GA 30342, USA

Treatment strategies for cancer have progressed greatly in recent decades. Since the approval of tamoxifen to modulate estrogen receptors (ER) in ER-positive breast cancer in the 1970s, improved understanding of gene expression changes in cancer have led to the development of effective, new targeted therapies [1]. Targeted therapies preferentially affect oncogenic cells, relatively sparing healthy tissue, reducing adverse effects found in other cancer therapies, such as chemotherapy or radiation [2]. However, even with the efficacy of established targeted therapies, cancers can develop resistance (ex: melanomas may become vemurafenib resistant, necessitating other inhibitors downstream of the BRAF signaling pathway) [3,4]. Because of this, new pathways and treatments must be elucidated to continually develop better therapies.

In the process of tumorigenesis, mutations in oncogenes and the loss of tumor suppressors give tumor cells a proliferative and survival advantage. Conversely, because of these altered profiles in genes’ expressions, they may have vulnerabilities that can be targeted. Targets can include the induction of tyrosine kinases, dependence on novel substrates, and altered metabolic profile [5,6,7]. This issue, “Targeting the Vulnerabilities of Oncogene Activation”, focuses on novel discoveries of signaling changes in tumorigenesis, as well as how treatments directed towards these potential targets show potential. Focusing on these specific vulnerabilities can guide the development of therapeutic agents, which can be used in both benign and malignant proliferative disorders.

The findings presented in this issue apply to multiple conditions, including breast cancer, gastric cancer, Sturge-Weber syndrome, and prostate cancer. However, because signaling mechanisms can be shared across different disorders, novel findings pertaining to one condition could benefit other conditions with the same vulnerability.

Felici et al. describe the efficacy of soluble fms-like tyrosine kinase 1 (sFLT-1) in inhibiting cancer growth and inducing tumor regression in a mouse model of ER-positive human breast carcinoma. In tumorigenesis, upregulated vascular endothelial growth factor (VEGF) contributes to angiogenesis, providing tumors with oxygen, nutrition, and growth factors. Through selective inhibition of VEGF with sFLT-1 (delivered via an adenovirus vector), treated mice had 91% decreased tumor size and 50% reduced vascular density compared to controls. Treatment led to the apoptosis of tumor vascular endothelial cells, followed by necrosis and fibrosis in the tumor, demonstrating how targeting angiogenesis pathways led to nutrition deprivation for the tumor. Furthermore, this mode of VEGF inhibition appeared to preferentially affect tumor cells, as there was no systemic delay in cutaneous wound healing [8]. Given resistance to ER targeted therapies in ER-positive breast cancer, inhibiting VEGF could serve as another tool in breast cancer and other neoplasms [9].

In another breast cancer study, Wang et al. found that ZNF582-AS1, a long non-coding RNA (lncRNA), is under-expressed in breast cancers. In particular, low ZNF582-AS1 expression is linked with high-grade or ER-negative tumors. ZNF582-AS1 is thought to have tumor suppressive properties, given its lowered expression in multiple malignancies, including colorectal and cervical cancer. Wang et al. found that lowered ZNF582-AS1 in breast cancer could be linked to promoter methylation. Through bioinformatics analysis, ZNF582-AS1 could downregulate the HER2-mediated pathway for breast cancer, or it can bind to has-miR-940, an oncogenic mRNA in breast cancer. Metabolic changes in breast cancer, such as upregulation in hypoxia inducible factor 1 (HIF-1), which is linked to tumor growth, could also contribute to the suppression of ZNF582-AS1. Understanding ZNF582-AS1 as a target could contribute to future cancer therapies that capitalize on its tumor suppressor activity [10].

Qi et al. present the elucidation of a novel TET1/FOXO4 to Wnt/β-Catenin signaling pathway in gastric cancer. In gastric cancer, Wnt/β-Catenin plays essential roles in the epithelial–mesenchymal transition of cancer cells, as well as the self-renewal of cancer stem cells. TET1/FOXO4 activity inhibits Wnt/β-Catenin effects, as FOXO4 and interaction with β-Catenin sequesters the latter to the cytoplasm to prevent transcription of Wnt target genes. In this study, the knockdown of TET1 led to increased metastasis, while overexpression suppressed gastric cancer metastasis. The discovery of these novel interactions present new therapeutic targets that can be used to prevent metastatic gastric cancer. Furthermore, TET1 and FOXO4 could become diagnostic markers for the prognosis of gastric cancer to better stratify risk for treatment [11].

Sasaki et al. describe a novel model for endothelial vascular malformations for conditions, such as Sturge-Weber syndrome, and they demonstrate that upregulation of certain targetable factors contribute to this phenotype. The study found that mutations in GNAQ contribute to vascular malformations and introduced this mutation to MS1 endothelial cells to create mouse models for evaluating the treatment of vascular diseases. Subsequently, Sasaki et al. found that C-Kit (CD117), which can be blocked by imatinib, was upregulated in this cell line. Subsequent treatment of mouse xenografts with the GNAQ mutation with imatinib led to reduced volume compared to controls. This marks imatinib as a potential treatment for Sturge-Weber syndrome and other conditions with a vascular malformation phenotype [12].

To clarify the effect of caffeic acid phenethyl ester (CAPE) on prostate cancer, Chang et al. elucidated specific targets of the compound and the subsequent downstream effects on signaling in prostate cancer. The study revealed that CAPE results in inhibition of the oncogene mucosa-associated lymphoid tissue 1 (MALT1), with subsequent inhibition of NF-κB activity, contributing to cell proliferation, invasion, and tumorigenesis. CAPE reduced MALT1 expression through inhibition of the androgen receptor (AR) or through inducing p53. CAPE also inhibited NF-κB in p53 and androgen receptor (AR)-negative prostate cancer cells, as CAPE binds NF-κB, directly preventing translocation to the nucleus. Chang et al. also presented a novel finding—that MALT1 is downregulated by p53. Meanwhile, CAPE also induced the ERK/JNK/p38/AMPKα1/2 pathway, but pretreatment with MAPK or AMPKα1/2 inhibitors did not interfere with the ability of CAPE to inhibit MALT1 activity. This study clarified key mediators in the CAPE signaling mechanism, which could help refine treatment considerations [13].

Overall, the findings in this special edition highlight how unique adaptations in cancer gene expression can also be vulnerabilities to be targeted in potential therapies. These findings span a range of conditions and demonstrate the potential for investigation into novel pathways in tumorigenesis.
